# Enhanced phosphorus elimination from aquatic systems employing modified granular waterworks derived sludge composite materials: Mechanistic evaluation and process optimization

**DOI:** 10.1371/journal.pone.0334439

**Published:** 2025-10-31

**Authors:** Liwenze He, Jintao Yan, Lin Wang, Jiaming Ni, Xi Li, Ping Li, Haiquan Li, Yu Chen, Ying Liu

**Affiliations:** 1 Department of Civil Engineering, Chengdu Technological University, Chengdu, China; 2 School of Materials and Environmental Engineering, Chengdu Technological University, Chengdu, China; 3 Faculty of Environmental Science and Engineering, Southwest Jiaotong University, Chengdu, China; National Chung Cheng University, Taiwan & Australian Center for Sustainable Development Research and Innovation (ACSDRI), AUSTRALIA

## Abstract

A novel granular waterworks-derived sludge composite (GT La-WDS) was synthesized via green and low-carbon hydrothermal carbonization combined with a calcination-free granulation method, demonstrating exceptional phosphate adsorption performance and potential as constructed wetland filler. Comprehensive characterization (SEM, XRF, BET, FTIR, XRD) revealed its hierarchical porous morphology, chemical composition, and hydroxyl/ligand-functionalized surfaces. Optimal phosphate adsorption capacity (20.11 mg/g) was achieved at pH 4, with adsorption mechanisms dominated by ligand substitution and formation of inner-sphere complexes, as supported by quasi-second-order kinetic modeling and Freundlich isotherm conformity. Dynamic column tests showed prolonged breakthrough (from 168 h to 432 h) and exhaustion times (from 588 h to 2088 h) with increasing bed heights (10 cm to 30 cm), achieving total adsorption capacities of 9.276 mg/g. Effluent phosphate concentrations remained below 0.5 mg/L (Chinese National Effluent Standard Class 1B) for 588 h, with sustained removal efficiency over 2088 h, indicating remarkable longevity. This sludge-derived composite presents a cost-effective solution for phosphorus sequestration, offering dual benefits of eutrophication mitigation and sustainable sludge valorization, with particular relevance to low-carbon constructed wetland systems.

## Introduction

Excessive phosphorus discharge from municipal wastewater into aquatic environments constitutes a primary driver of eutrophication in receiving water bodies [1]. Excessive phosphorus loading surpassing 0.015 mg/L in lentic ecosystems has been established as the primary trigger for eutrophication onset [2], underscoring the necessity of phosphorus abatement as an essential management approach for controlling algal proliferation [3]. Current predominant phosphorus removal strategies in wastewater treatment encompass polyphosphate-accumulating organism (PAO)-mediated bioprocesses, metal salt-induced coagulation, and sorbptive sequestration mechanisms [4]. While microbial-mediated phosphorus elimination is acknowledged as an economically viable approach, its operational scalability remains intrinsically limited by the stringent environmental controls and precise process parameters essential for maintaining the metabolic functionality of polyphosphate-accumulating microbial consortia [5]. Although chemical precipitation rapidly and efficiently removes phosphorus, its use is constrained by high coagulant demand, challenging sludge handling, and secondary pollution risks [6,7]. Adsorption-based phosphorus removal has garnered extensive application in treating diverse phosphate-laden wastewater streams due to its merits including simplicity of implementation, operational flexibility, high cost-effectiveness, stable adsorption-desorption performance, and regeneration feasibility [8,9].

Recent advances have demonstrated the valorization of diverse solid wastes—including hydrotalcite [10], red mud [11], and water treatment sludge [12]—as precursors for cost-effective phosphate adsorbent synthesis, achieving dual benefits of waste upcycling and reduced material fabrication expenditures. Waterworks-derived sludge (WDS), generated as residual solids of aluminum-based coagulation processes in potable water purification facilities, represents the most prevalent residual material in potable water treatment processes [13]. With valorization techniques still under development, WDS is predominantly landfilled, leading to its low resource utilization rate [14]. Characterization studies reveal that WDS, predominantly composed of iron and aluminum salts, exhibits high phosphate uptake performance due to increased interfacial contact area and mesoporous structure, thereby allowing its use as an affordable sorbent substance for efficient phosphorus extraction during wastewater remediation processes [15,16]. However, the inherently constrained phosphate adsorption capacity of WDS necessitates targeted modification strategies to augment its phosphorus sequestration efficiency [17]. Lanthanum (La), a chemically stable and geologically abundant rare earth element, possesses strong affinity and selective binding capability for phosphate. It can undergo chemical precipitation and ligand exchange with phosphate to form surface precipitates and inner-sphere complexes, thereby achieving phosphate removal. Therefore, lanthanum can be used to modify adsorbents to enhance their phosphorus removal performance [18]. Chen et al. [19] successfully immobilized lanthanum onto WDS surfaces for phosphorus adsorption, demonstrating its enhanced adsorption performance with the phosphate adsorption capacity increasing from 6.19 mg/g to 24.45 mg/g after lanthanum modification.

Artificial marshland configurations, serving as a novel environmentally sustainable solution for effluent management and aquatic system revitalization, have attained global adoption across water quality improvement sectors [20]. The elimination of phosphorus in engineered wetland systems principally occurs through vegetative assimilation, precipitation, substrate adsorption, and microbial degradation. Among these, phosphorus adsorption and retention by substrates represent critical pathways for inflow phosphorus in wetlands [21,22]. Consequently, research on adsorptive substrates has garnered considerable scientific interest. Adsorbents are predominantly used in powdered form due to their effective adsorption capacity [23]; however, they face practical limitations such as difficult filtration, poor separation from water, and notable constraints in constructed wetlands, making them unsuitable as fillers. In contrast, granular fillers utilize their larger particle size and superior mechanical strength to enhance solid-liquid separation efficiency. They preserve the original adsorption performance of the adsorbent materials while avoiding issues such as difficult recovery, problematic separation, and a tendency to cause clogging in equipment. Studies have shown that the use of granular fillers can effectively maintain system hydraulic conductivity, extend operational cycles, and reduce maintenance requirements, better meeting the practical operational demands of constructed wetlands [24]. The calcination-free granulation technique involves water vapor curing to activate granule reactivity, achieving binder-free particle consolidation. Compared with conventional high-temperature calcination, this approach retains mechanical strength while preserving granular architecture and significantly reduces fabrication energy consumption [25]. Hydrothermal carbonization is a sludge carbonization technology that converts organic matter into biochar under specific temperature and autogenous pressure conditions within a closed aqueous system. Hydrothermal carbonization, characterized by low energy consumption, mild reaction conditions, and operational simplicity, has been extensively applied to municipal sludge resource recovery [26]. This study integrates hydrothermal carbonization with calcination-free granulation to fabricate modified sludge granules, evaluating their phosphate adsorption performance and applicability as constructed wetland fillers, thereby establishing a low-pollution, energy-efficient green pathway for waterworks-derived sludge valorization.

## Methodological framework and material characterization

### Preparation methodologies for investigative media

WDS specimens were sourced from partially dewatered sludge residues at a public water supply plant in Chengdu, demonstrating a basal moisture concentration of 82.12 ± 0.50%. The sludge underwent natural air-drying under ambient conditions for 48 hours, followed by dehydration in an oven maintained at 60 °C until achieving consistent mass. Subsequently, the dried material was mechanically pulverized and passed through a standard 100-mesh sieve. The processed samples were ultimately preserved in hermetically sealed polyethylene containers for subsequent analysis.

La-modified WDS (La-WDS) was prepared by modifying WDS using the lanthanum immobilization method previously developed by Chen et al. [19] to enhance phosphate adsorption capacity. The composite suspension was formulated by incorporating 3 g of untreated WDS particulate matter into 27 mL of 0.2 M lanthanum chloride aqueous solution. This precursor mixture was subsequently subjected to dropwise introduction of 3 mL concentrated sodium hydroxide solution (12 M) while maintaining constant agitation using a magnetic stirrer over a 30-minute homogenization period. The precursor suspension was loaded into a Teflon-lined stainless steel hydrothermal vessel and thermally processed at 170 °C under autogenous pressure for a duration of 3 hours. Post-synthesis, the crystalline precipitate was isolated through Buchner filtration apparatus, sequentially rinsed with triplicate aliquots of Type I ultrapure water, then dehydrated in a convection oven at 60 °C until mass stabilization. The desiccated particulates underwent mechanical size classification employing certified 100-mesh brass sieves, yielding the target La-WDS composite material for subsequent characterization.

La-WDS was granulated via a calcination-free ceramsite preparation method. GT La-WDS granules were prepared by thoroughly mixing La-WDS, cement, fly ash additive, and an activator (quicklime: gypsum = 1:1) at specified ratios. For every 100 g of raw materials, 30 ~ 35 mL of water and 2 g of sodium silicate were added, followed by granulation to achieve a particle size range of 3 ~ 5 mm. The particulate matter underwent sequential processing involving 2 hours of ambient curing, 12 hours of hydrothermal treatment through vapor-phase saturation, followed by thermal equilibration and desiccation to yield GT La-WDS composite granules.

### Material evaluation methodologies and instrumental analysis

The microstructural features and surface architecture of GT La-WDS were analyzed through SEM using a ZEISS Sigma 300 (Germany). The predominant elemental constituents were quantified through XRF utilizing a Thermo Fisher Scientific ARL Perform’X analytical system (USA). The BET surface area and porosity parameters were evaluated via nitrogen adsorption-desorption isotherms measured on a Micromeritics ASAP 2460 gas sorption analyzer (USA). Surface chemical functionalities of GT La-WDS were spectroscopically profiled via FTIR analysis (Thermo Fisher Nicolet iS5, USA), with spectral acquisition spanning the 400 ~ 4000 cm^-1^ wavenumber range to identify characteristic bond vibrations. The crystallographic properties of GT La-WDS were analyzed using a Rigaku Ultima IV X-ray diffractometer (Japan) to resolve its phase composition. The structural degradation rate and combined fracture-attrition indices of GT La-WDS were evaluated in compliance with the Chinese National Construction Standard CJ/T 299–2008, which governs synthetic ceramsite filtration media for aqueous treatment applications (Table S1 in S1 File) [27].

### Adsorption experiments

The adsorption characteristics of GT La-WDS in aqueous solutions were systematically investigated through parametric studies, kinetic experiments, isotherm analyses, and thermodynamic evaluations.

#### Effect of influencing factors.

Adsorption trials were performed in 100 mL conical flasks under controlled conditions using a thermostatic orbital shaker (THZ-98A, Shanghai Yiheng Scientific Instrument, 180 rpm), with the following experimental parameters:

(1) Effect of initial pH: GT La-WDS was administered at a dosage of 2 g/L into 100 mL conical flasks charged with a 20 mg/L phosphorus solution. The pH values were systematically calibrated to 3, 4, 5, 6, 7, 8, and 9 using 0.1 M HCl or NaOH, followed by a 720-minute adsorption phase at 30°C under continuous agitation (180 rpm).
(2) Effect of dosage: GT La-WDS was added at varying dosages (0.1, 0.5, 1, 2 g/L) to conical flasks containing 20 mg/L phosphorus solution, followed by a 720 min adsorption process at 30 °C and the optimal pH under continuous shaking (180 rpm).
(3) Effect of coexisting ions: GT La-WDS was dosed at 2 g/L into conical flasks containing 20 mg/L phosphorus solution, with ionic solutions (NaCl, NaNO_3_, Na_2_SO_4_, Na_2_CO_3_) added at gradient concentrations of 0.1, 0.5, and 1 M. The adsorption process proceeded for 720 min at 30 °C and the optimal pH under continuous shaking (180 rpm).

The phosphorus elimination efficacy (R) and sorption capacity (q_t_) of GT La-WDS were derived through the computational application of Equations (1) and (2):


$$R=\frac{\left(C_{0}-C_{t}\right)}{C_{0}}\times 100\%$$ (1)

$$q_{t}=\frac{\left(C_{0}-C_{t}\right)\cdot V}{m}$$ (2)

with C_0_ and C_t_ characterizing the baseline and temporally resolved phosphorus concentrations (mg/L), V representing the total liquid phase volume (L), and m quantifying the mass of the GT La-WDS sorbent medium (g).

#### Adsorption kinetics.

In the adsorption trials, a 2 g/L aliquot of GT La-WDS was introduced into 100 mL Erlenmeyer flasks charged with a 20 mg/L phosphorus solution, subjected to sustained orbital agitation (180 rpm) at 30 °C to activate the sorption mechanism. The aqueous-phase phosphorus concentrations were monitored at predetermined temporal intervals (0, 15, 30, 45, 60, 120, 240, 360, 480, 720, 1440, 2160, and 2880 minutes) to quantify adsorption kinetics.

#### Adsorption thermodynamics.

GT La-WDS was dosed at 2 g/L into 100 mL conical flasks containing phosphorus solutions with initial concentrations spanning 20 ~ 80 mg/L (in 10 mg/L increments), followed by a 12 h adsorption process at 30 °C under continuous shaking (180 rpm). The equilibrium phosphate concentrations were quantified, with subsequent thermodynamic analysis of adsorption data performed through application of Langmuir and Freundlich isothermal models.

### Phosphorus fractionation analysis

Phosphorus fractionation in sediments includes loosely adsorbed phosphorus (NH_4_Cl-P), reducible iron-associated phosphorus (BD-P), aluminum-complexed phosphorus (NaOH-P), calcium-linked phosphorus (HCl-P), and refractory residual phosphorus (Res-P) [28]. Phosphorus speciation analysis of immobilized phosphorus on the adsorbent provides critical insights into the adsorption mechanisms governing phosphorus sequestration. Phosphorus species adsorbed onto GT La-WDS were analyzed using a sequential chemical extraction protocol (Table S2 in S1 File) [29].

### Dynamic adsorption of phosphate by GT La-WDS

Dynamic adsorption experiments were conducted by continuously passing phosphorus-containing wastewater through an adsorption column in a top-down direction, with flow regime controlled by a peristaltic pump. This configuration ensured sufficient contact between P in the solution and GT La-WDS, thereby validating the feasibility of GT La-WDS as a synthetic marshland medium for phosphate retention.

The phosphorus removal process is quantified through breakthrough curve analysis, established by graphing the normalized concentration ratio (C_t_/C_0_) as a function of temporal duration, wherein C_t_ represents the phosphorus concentration in effluent streams at specific time intervals and C_0_ indicates the baseline phosphorus concentration prior to treatment. The breakthrough concentration was defined according to the Grade 1B total phosphorus effluent limit (1 mg/L) mandated under Chinese National Standard GB 18918−2002 [30]. The breakthrough point was defined as C_t_/C_0_ = 0.1, and the exhaustion point was defined as C_t_/C_0_ = 0.9. The breakthrough time corresponded to the time at C_t_/C_0_ = 0.1, while the exhaustion time was determined at C_t_/C_0_ = 0.9.

## Results and discourse

### Structural and functional characterization of GT La-WDS

#### FT-IR spectroscopic investigation.

**Fig 1:**
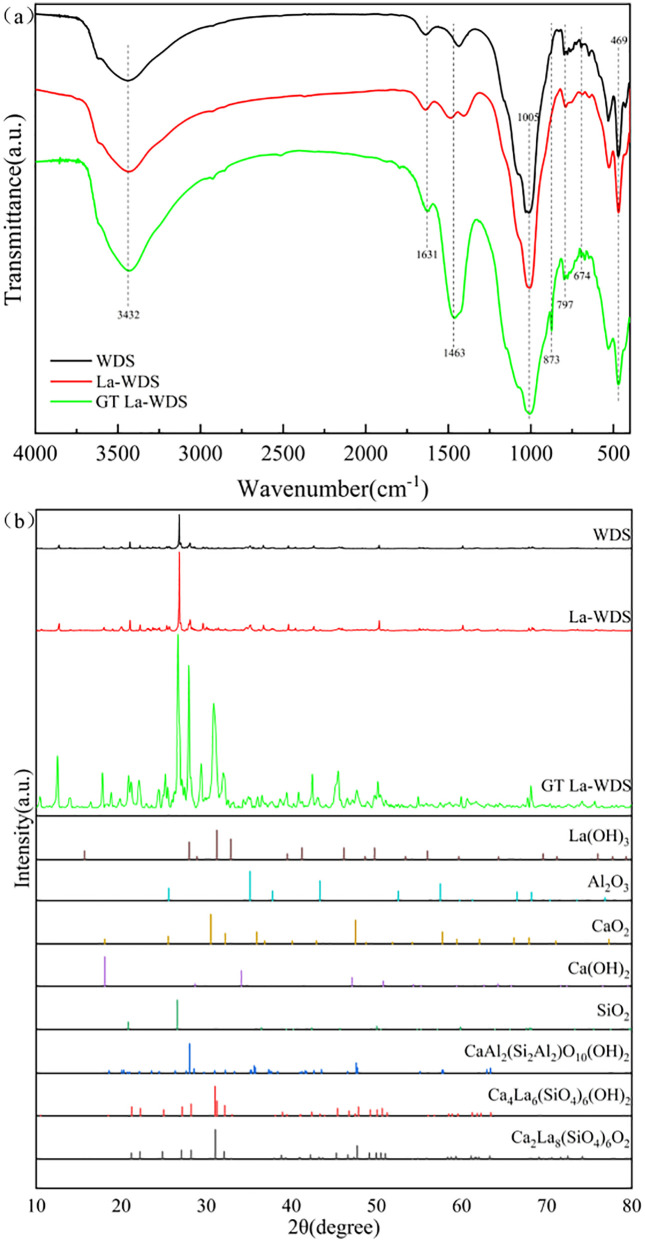
Comparative characterization of (a) FT-IR spectral profiles and (b) XRD patterns for the GT La-WDS.

Fig 1(a) presents the FT-IR profiles of WDS, La-WDS, and GT La-WDS. The absorption peaks at 3432 and 1631 cm^-1^ correspond to the O-H stretching vibration and bending vibration, respectively [31], demonstrating hydroxyl functional groups in WDS, La-WDS, and GT La-WDS. The characteristic bands detected at 1005 and 469 cm^-1^ correspond to the symmetric stretching and angular deformation modes of Si-O-Si linkages, respectively [32], demonstrating the presence of siloxane tetrahedral structures within the particles. The spectral signature at 797 cm^-1^ is ascribed to the angular bending mode of metal hydroxide groups (e.g., Al-OH) [33], while the peak at 674 cm^-1^ is assigned to the stretching vibration of La-O bonds [34]. The emergence of a new peak at 1463 cm^-1^ in GT La-WDS corresponds to O-Ca-O vibrational modes. The characteristic bands of C-S-H, including O-H, O-Ca-O, and Si-O-Si vibrations, provide conclusive evidence for the crystallization of C-S-H gel through hydration reactions triggered by cement additives in GT La-WDS [35].

Fig 1(b) displays a comparative XRD analysis of La-WDS and GT La-WDS. The raw material La-WDS is primarily composed of La(OH)_3_, Al_2_O_3_, and SiO_2_. In contrast, GT La-WDS not only retains these phases but also exhibits distinct diffraction peaks corresponding to CaO_2_ and Ca(OH)_2_, confirming the successful incorporation of calcium during the granulation process. Furthermore, several newly formed crystalline phases—including CaAl_2_(Si_2_Al_2_)O_10_(OH)_2_, Ca_4_La_6_(SiO_4_)_6_(OH)_2_, and Ca_2_La_8_(SiO_4_)_6_O_2_—are observed, indicating that a portion of Al, La, and Ca participated in chemical reactions leading to the formation of more complex and stable mineral structures. These newly generated phases, along with the remaining active components such as La(OH)_3_,Al_2_O_3_,CaO_2_ and Ca(OH)_2_, collectively contribute to the high phosphorus removal activity of GT La-WDS.

#### Surface morphology and pore structure analysis.

**Fig 2:**
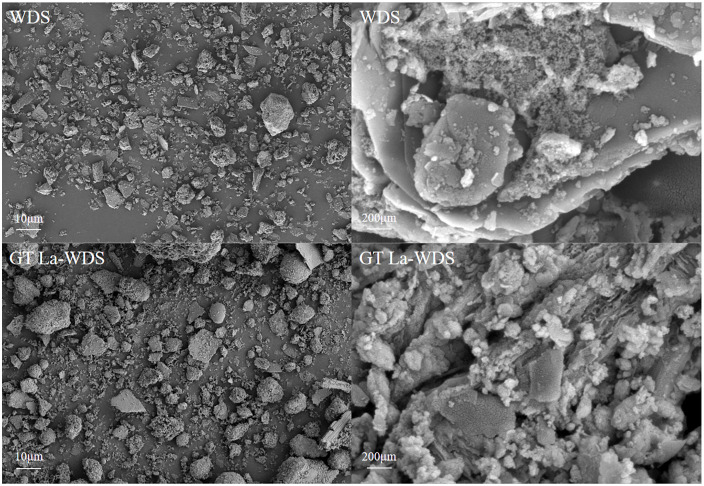
SEM images of WDS, La-WDS and GT La-WDS.

The SEM micrographs for WDS and GT La-WDS are presented in Fig 2. WDS displays a rough and uneven surface morphology dominated by flake-like structures. Following modification, the GT La-WDS surface exhibited roughened textures with protuberant structures, accompanied by stacked granular aggregates and surface-developed porous architecture, collectively contributing to an enhanced specific surface area. Compared to WDS, GT La-WDS exhibits a significantly rougher surface morphology with a well-developed porous architecture, indicative of enhanced surface textural properties.

**Fig 3:**
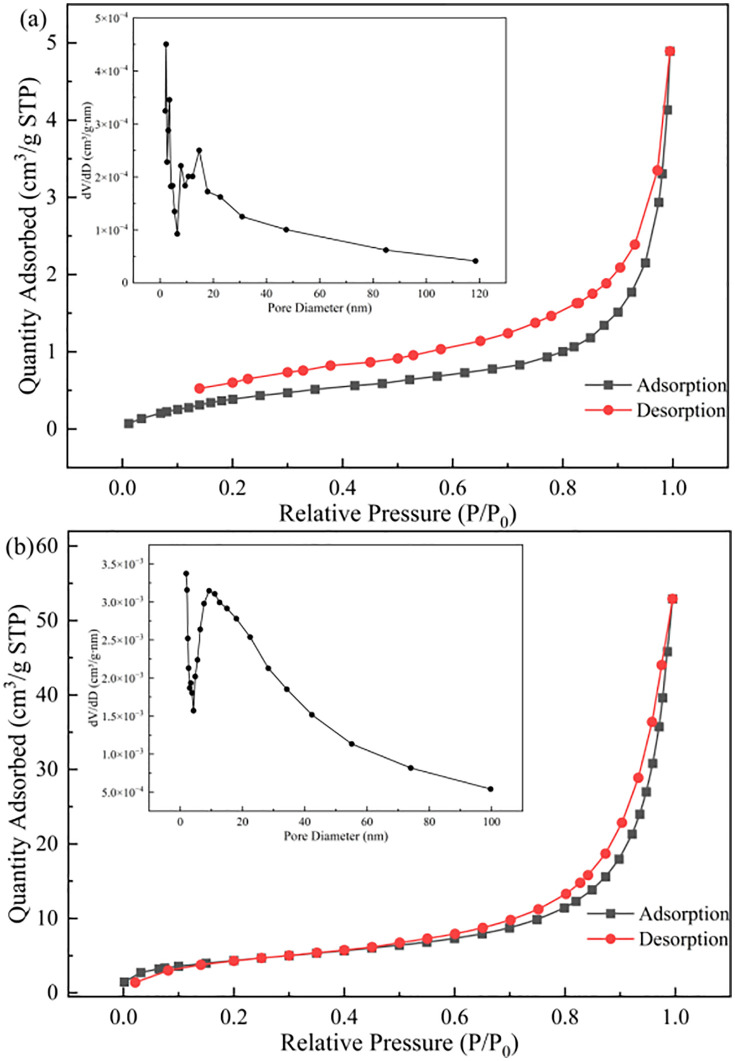
N_2_ adsorption-desorption isotherms and pore diameter distribution of (a) WDS and (b) GT La-WDS.

As shown in Fig 3, the N_2_ adsorption-desorption isotherms and pore size distribution of WDS before and after modification are presented. The adsorption capacity gradually increases with rising relative pressure, and it can be observed that GT La-WDS exhibits significantly higher adsorption capacity than raw WDS. The N_2_ adsorption-desorption isotherms of the materials conform to the type IV isotherm characteristic, indicating a predominantly mesoporous structure, which is further confirmed by the pore size distribution plot [36]. As evidenced by the pore structure data in Table 1, GT La-WDS exhibits a significantly enhanced specific surface area (15.30 m^2^/g) compared to raw WDS (1.79 m^2^/g), accompanied by increased pore volume and enlarged pore size distribution. This indicates that granulation significantly enhanced the SSA of GT La-WDS compared to WDS, with optimized porous architecture providing abundant active sites for phosphate adsorption, thereby improving phosphorus sequestration efficiency [37].

**Table 1:** Material architecture indicators.

Materials	Total pore capacity/(cm^3^·g^-1^)	Mean pore size/(nm)	Specific surface area/(m^2^·g^-1^)
**WDS**	0.006	16.16	1.79
**GT La-WDS**	0.082	20.94	15.30

#### Elemental analysis and performance evaluation.

The major elemental compositions of WDS and GT La-WDS are summarized in Table 2. GT La-WDS primarily comprises Si, Al, Ca, Fe, and K, consistent with the elemental profile of raw WDS. The significant La content in GT La-WDS, attributed to the lanthanum modification treatment, confirms the successful immobilization of La onto the granulated material surface. Concurrently, the Ca content in GT La-WDS increased from 6.71% (WDS) to 13.18%, attributed to the incorporation of Ca-containing compounds in granulation reagents.

**Table 2:** XRF results.

Samples	Si	Al	Ca	La	Fe	K	Na	Mg
**WDS**	35.89	21.33	6.71	0	11.48	5.17	1.63	1.16
**GT La-WDS**	34.27	15.79	13.18	11.88	4.41	1.82	1.76	1.54

The performance parameters of GT La-WDS were assessed in compliance with the Chinese Urban Construction Industry Standard CJ/T 299–2008 Engineered Ceramic Granular Filtration Media for Municipal Water Purification Systems, with key metrics summarized in Table 3. The sum of breakage and abrasion rates of the granules was determined to be 0.42%, significantly lower than the 6% threshold stipulated by the CJ/T 299–2008 standard, while the disintegration rate measured 3.68%. These results demonstrate superior mechanical strength, confirming compliance with the requirements for artificial ceramsite filter media and validating their applicability as constructed wetland fillers.

**Table 3:** Performance indicators of GT La-WDS.

Measurement indicators	Measured value	Artificial Ceramsite Filter Media for Water Treatment (CJ/T 299–2008)
**Breakup rate/(%)**	3.68	/
**Total of breakage and wear rates/(%)**	0.42	≤6

### Phosphate adsorption behavior of GT La-WDS

#### Effect of solution pH.

**Fig 4:**
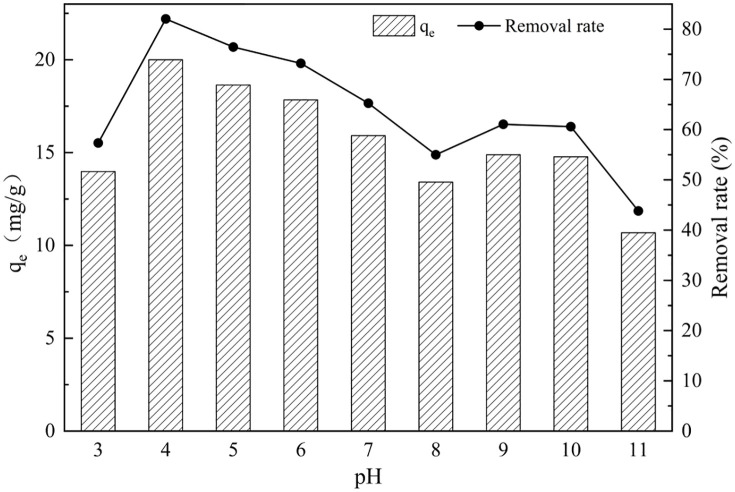
pH influence on phosphorus uptake.

**Fig 5:**
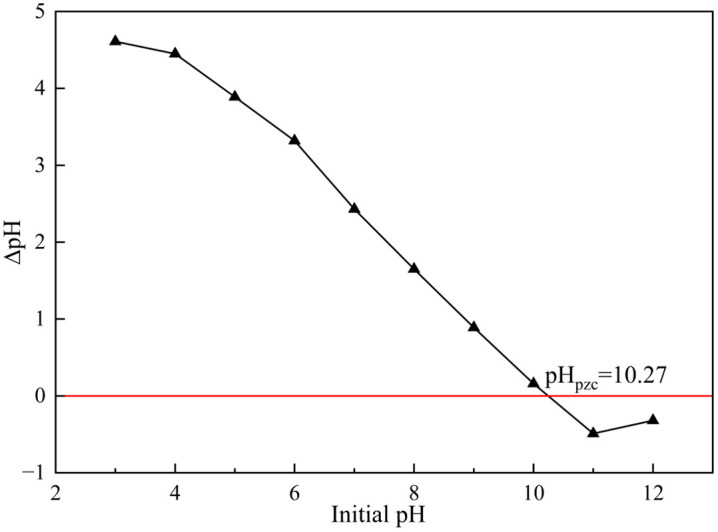
Zeta potentials of GT La-WDS.

The effect of solution pH on phosphate adsorption by GT La-WDS is shown in Fig 4. The adsorption capacity for phosphate was optimized under acidic conditions, peaking at 20.11 mg/g at pH 4. A gradual decline in adsorption performance was observed with increasing pH, likely governed by surface electrical properties of GT La-WDS and ionization/hydrolysis behavior of phosphate species [38]. Within the pH range of 2–7, phosphate predominantly exists as H_2_PO_4_^-^ in aqueous solutions, while HPO_4_^2-^ becomes the dominant species in the pH range of 7–12. The La species immobilized on GT La-WDS surfaces exhibit strong affinity toward H_2_PO_4_^-^, which facilitates preferential adsorption via ligand-exchange mechanisms [19]. The point of zero charge (pHpzc) of GT La-WDS was determined according to the referenced method (Table S3 in S1 File), and was found to be 10.27 from Fig 5 [39]. When the solution pH is lower than the pHpzc, the surface of GT La-WDS is positively charged, and electrostatic attraction favors the adsorption of phosphate anions. Conversely, when the solution pH exceeds the pHpzc, the GT La-WDS surface becomes negatively charged, leading to a decrease in phosphate adsorption capacity due to electrostatic repulsion [40]. Under alkaline conditions, the abundant hydroxyl ions in solution compete for active sites on GT La-WDS, further reducing its adsorption capacity [41]. The calcium Ca content in granulation additives facilitated Ca^2+^ leaching under alkaline conditions, enabling precipitation of hydroxyapatite with phosphate ions, which enhanced phosphate adsorption by GT La-WDS at pH 8–10 [42]. Synthesizing these effects, the optimal pH was identified as 4.

#### Effect of adsorbent dosage.

**Fig 6:**
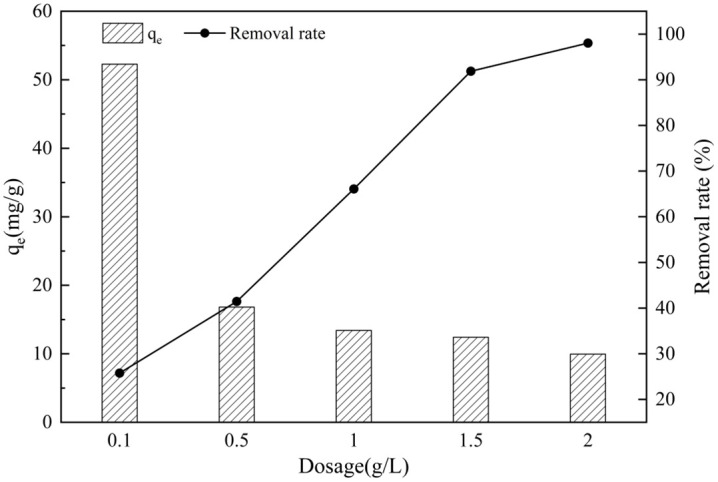
Dosage influence on phosphorus uptake.

The influence of GT La-WDS loading levels on phosphorus adsorption performance is depicted in Fig 6. Upon escalating GT La-WDS input from 0.1 to 2 g/L, the phosphate removal efficiency rose from 18.41% to 97.47%. This enhancement stems from the rise in accessible adsorption sites relative to a fixed phosphate concentration in solution. However, the adsorption capacity exhibited a declining trend. This is likely because, under the condition of a fixed initial phosphate concentration, increasing the dosage of GT La-WDS accelerates phosphate removal, leading to a rapid decrease in the remaining phosphate concentration in the solution and a reduction in the mass transfer driving force for adsorption. Furthermore, at higher dosages, the effective active sites available for adsorption per unit mass of GT La-WDS cannot be fully utilized, resulting in a decrease in the adsorption capacity of GT La-WDS for phosphate [43].

#### Adsorption kinetics.

**Fig 7:**
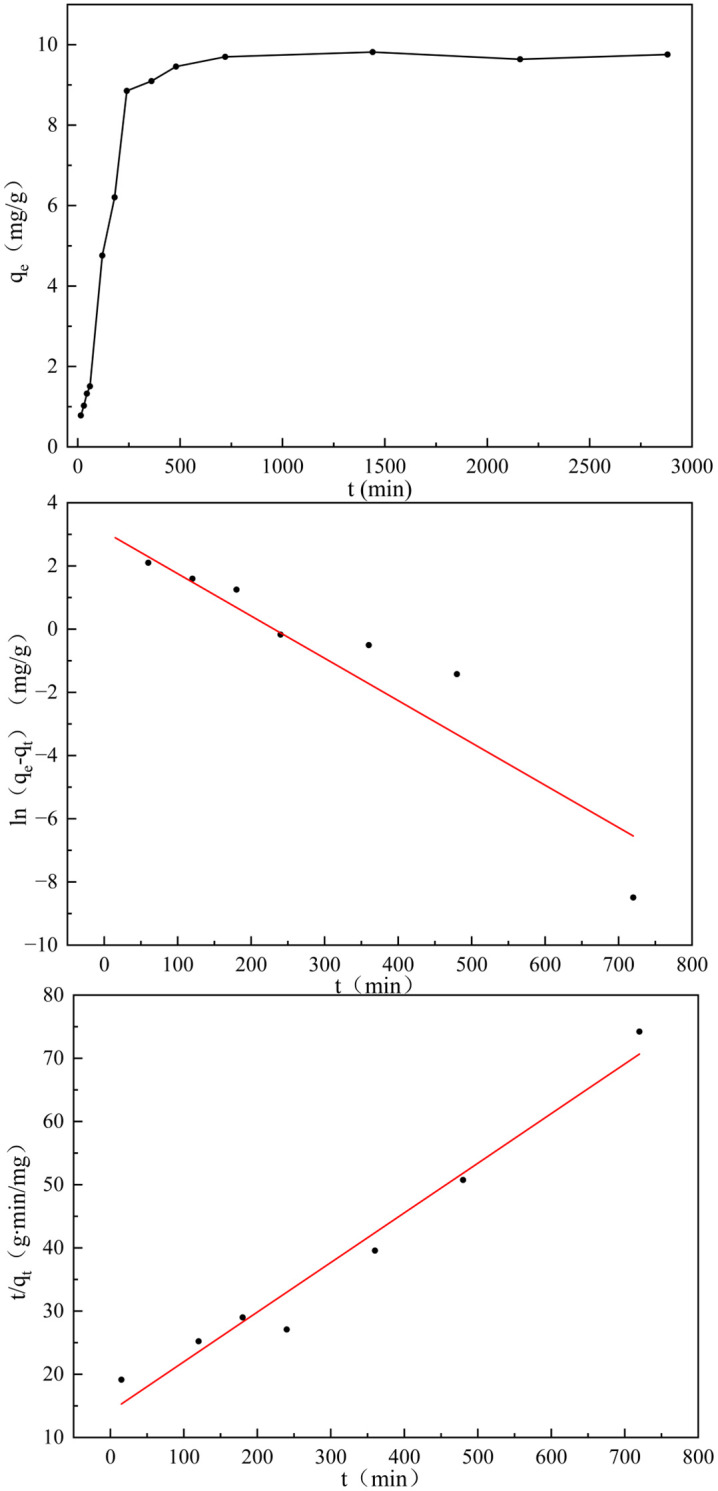
Adsorption kinetic curves of phosphate.


The adsorption kinetics were investigated through application of pseudo-first-order (Equation 3) and pseudo-second-order (Equation 4) dynamic models, with corresponding regression analyses illustrated in Fig 7 and summarized in Table 4.

**Table 4:** Kinetic parameters for phosphate adsorption of GT La-WDS.

Pseudo-first-order kinetic model			Pseudo-second-order kinetic model		
*k*_*1*_	*q*_*e,cal*_	*R* ^*2*^	*k*_*2*_	*q*_*e,cal*_	*R* ^*2*^
0.0148	43.116	0.8944	0.0004	12.739	0.9661

$$ln\left(q_{e}-q_{t}\right)=lnq_{e}-k_{1}t$$ (3)

$$\frac{t}{q_{t}}=\frac{\text{1}}{k_{2}{q_{e}}^{2}}+\frac{t}{q_{e}}$$ (4)

with q_e_ and q_t_ represent the equilibrium adsorption performance and time-dependent adsorption performance (mg/g) of the adsorbent. The parameters k_1_ and k_2_ respectively quantify the reaction rate coefficients (1/min) for the pseudo-primary and pseudo-secondary kinetic frameworks.

The fitting results demonstrate that during the initial adsorption phase, GT La-WDS exhibited abundant surface reactive loci, inducing exponential growth of phosphorus uptake capacity. Adsorption equilibrium was achieved within 720 min, yielding a capacity of 9.699 mg/g. The pseudo-second-order kinetic counterpart demonstrated a significantly higher determination coefficient (R^2^ = 0.9661) exceeding that of the pseudo-first-order counterpart. The calculated equilibrium uptake (q_e,cal _= 12.739 mg/g) closely approximates the experimental value (q_e _= 9.699 mg/g), confirming chemisorption-dominated phosphate adsorption by GT La-WDS as the primary rate-limiting factor [44], with the pseudo-second-order kinetic model providing the most accurate description of the adsorption process.

### Adsorption thermodynamics

**Fig 8:**
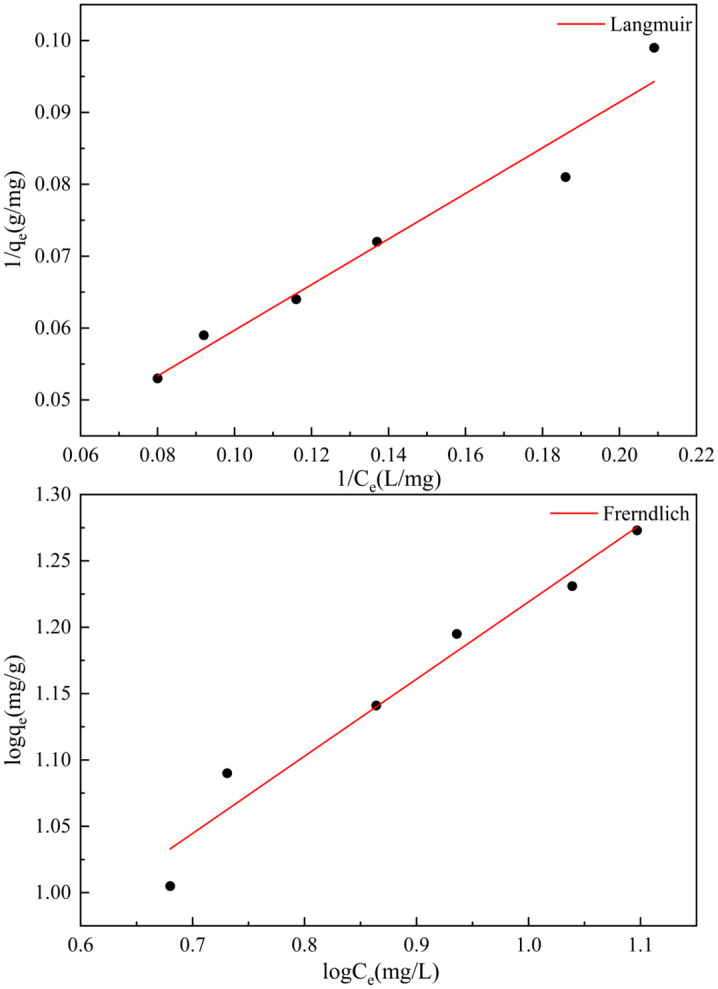
Isothermal and thermodynamic adsorption curves.


Fig 8 presents the Langmuir (Equation 5) and Freundlich (Equation 6) adsorption isotherms for phosphate adsorption by GT La-WDS, with the corresponding model parameters summarized in Table 5.

**Table 5:** Phosphate adsorption isotherm and thermodynamic parameters.

T/°C	Langmuir			Freundlich		
	*k*_*l*_	*q*_*max*_	*R* ^*2*^	*kf*	*1/n*	*R* ^*2*^
**25**	0.0115	49.020	0.9529	0.9702	0.7629	0.9760

$$\frac{\text{1}}{q_{e}}=\frac{\text{1}}{q_{m}}+\frac{\text{1}}{q_{m}k_{l}C_{e}}$$ (5)

$$lnq_{e}=k_{f}+\frac{\text{1}}{n}\ln C_{e}$$ (6)

with C_e_ is the residual phosphorus concentration (mg/L) at equilibrium; q_e_ and q_m_ characterize the operational uptake capacity and theoretical monolayer saturation threshold (mg/g) of the adsorbent, respectively. The k_l_ and k_f_ respectively signify the Langmuir affinity parameter and Freundlich adsorption intensity constant, with n is the Freundlich heterogeneity index.

Both isotherm models demonstrated excellent applicability with high goodness-of-fit in describing the phosphate uptake process by GT La-WDS. The Langmuir model assumes that adsorption occurs in a monolayer on a homogeneous surface with no interaction between adsorbed molecules. In contrast, the Freundlich model is applicable for describing multilayer adsorption processes on heterogeneous surfaces, assuming an exponential distribution of site energies and allowing for interactions between adsorbate molecules [45]. The Freundlich model exhibited a better fit (R^2^ = 0.9760) compared to the Langmuir model (R^2^ = 0.9529). These results strongly indicate that the Freundlich model more accurately describes the phosphate adsorption process by GT La-WDS, confirming that phosphate adsorption proceeds through multilayer adsorption mechanisms on heterogeneous surfaces. The parameter 1/n of the Freundlich model is indicative of adsorption characteristics. Values of 1/n > 2 indicate unfavorable adsorption, while 1/n < 1 suggests beneficial uptake processes [46]. The obtained 1/n value (<1) in this study confirms the highly favorable phosphate adsorption by GT La-WDS.

### Impact of coexisting anions

**Fig 9:**
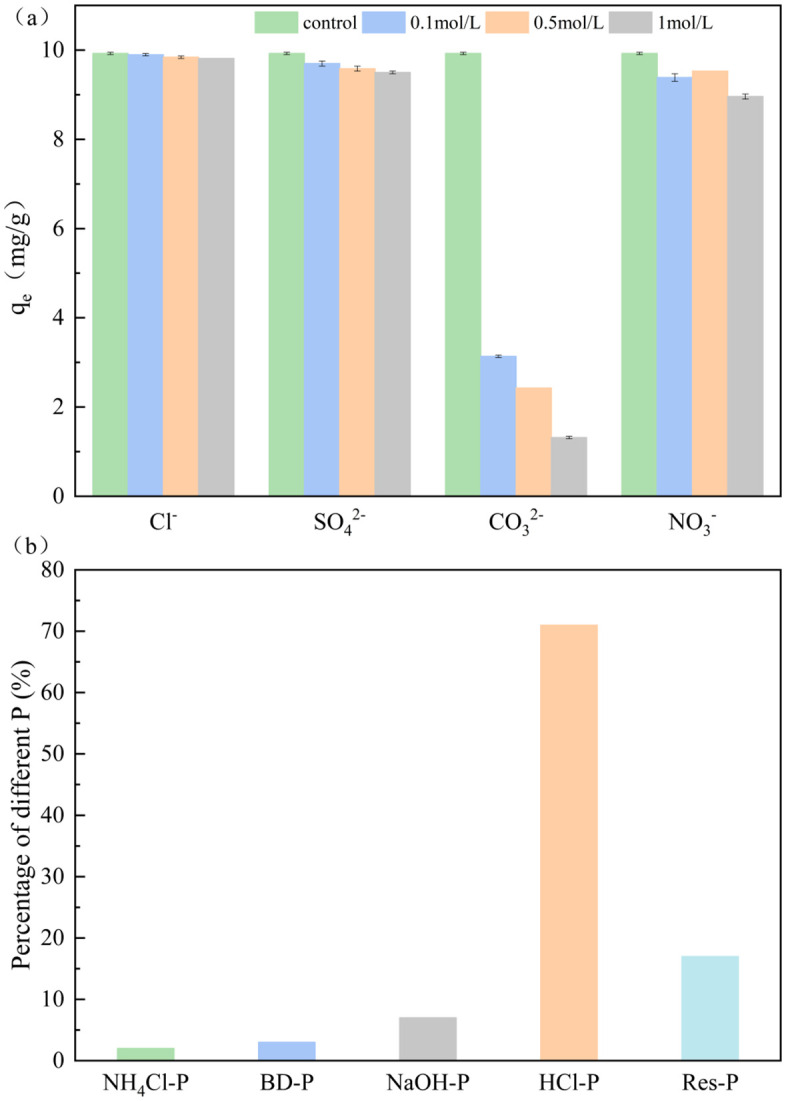
Coexisting ions and phosphorus species.

Fig 9(a) illustrates the influence of coexisting anions on phosphate uptake by GT La-WDS. As shown in Fig 9(a), Cl^-^, SO_4_^2-^, and NO_3_^-^ exhibited negligible interference with phosphate removal, whereas CO_3_^2-^ demonstrated significant inhibitory effects on GT La-WDS-mediated phosphate adsorption. At a CO_3_^2-^ concentration of 1 M, the phosphate uptake performance sharply declined from 9.926 to 1.318 mg/g, likely due to competitive occupation of surface adsorption sites by CO_3_^2-^, which impeded phosphate binding to GT La-WDS through ligand-exchange mechanisms. Furthermore, hydrolysis of CO_3_^2-^ elevates solution pH to the range of 11-11.5, which is above the pHpzc of GT La-WDS (10.27). Under these conditions, the adsorbent surface becomes negatively charged, weakening the electrostatic attraction between the adsorbent and anionic phosphate species and thereby reducing the adsorption capacity. This observation demonstrates the critical role of electrostatic interactions in GT La-WDS-mediated phosphate adsorption [47].

### Phosphorus fractionation and immobilization mechanisms

The fractionation results of phosphate species adsorbed onto GT La-WDS via sequential chemical extraction are presented in Fig 9(b). Loosely adsorbed phosphorus (NH_4_Cl-P) and reducible iron-associated phosphorus (BD-P) constitute the predominant labile phosphorus fractions, which are readily mobilized into aquatic systems under environmental perturbations [48]. Aluminum-complexed phosphorus (NaOH-P), calcium-linked phosphorus (HCl-P), and refractory residual phosphorus (Res-P) are classified as stable phosphorus fractions [49]. In the phosphorus sequestered by GT La-WDS, loosely adsorbed phosphorus (NH_4_Cl-P), reducible iron-associated phosphorus (BD-P), aluminum-complexed phosphorus (NaOH-P), calcium-linked phosphorus (HCl-P), and residual phosphorus (Res-P) accounted for 2%, 3%, 7%, 71%, and 17% of the total sequestered phosphorus, respectively. HCl-P accounted for the highest proportion of the total sequestered phosphorus, attributed to the abundant Ca and La in GT La-WDS, where phosphorus bound to Ca/La predominantly exists as HCl-P [50]. Calcium oxides and hydroxides immobilize phosphorus through hydroxyapatite precipitation via chemical precipitation [42], while lanthanum oxides and hydroxides fix phosphorus by forming inner-sphere complexes with phosphate through ligand-exchange mechanisms [51]. Furthermore, GT La-WDS contains substantial Al content, where aluminum oxides and hydroxides immobilize phosphorus as NaOH-P via ligand-exchange mechanisms [37].

It can be concluded that the main mechanisms of phosphorus adsorption by GT La-WDS may involve electrostatic attraction, ligand exchange, and chemical precipitation. Under acidic conditions, combined with the influence of pH, phosphate interacts with Al and La in GT La-WDS via electrostatic interaction and ligand exchange, forming inner-sphere complexes. Under alkaline conditions, Ca in GT La-WDS is released into the solution and reacts with phosphate to form hydroxyapatite precipitates, thereby removing phosphate from the solution.

### Dynamic adsorption of phosphate by GT La-WDS

**Fig 10:**
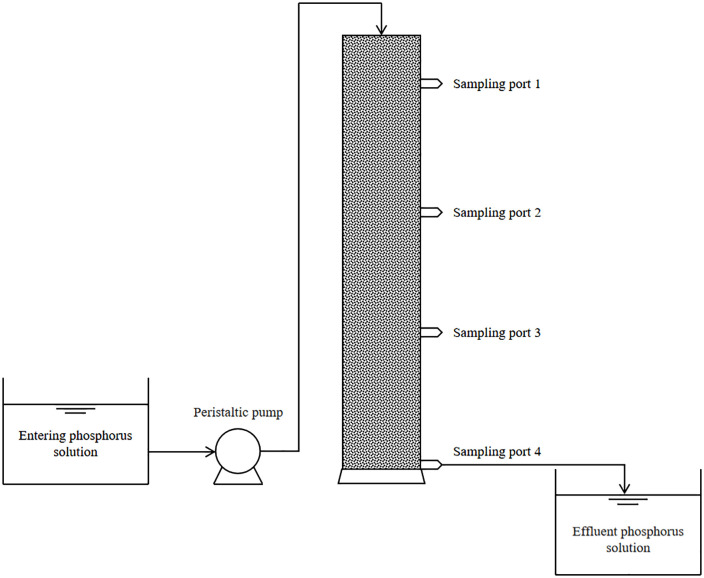
GT La-WDS dynamic adsorption column apparatus diagram.

The dynamic adsorption column setup of GT La-WDS is shown in Fig 10, with the phosphorus solution flowing at 5 mL/min and containing 10 mg/L phosphate, pH 4, with a water residence period of 1 h. The discharged phosphorus level was measured every 12 hours.

#### Breakthrough curves at varying bed heights.

**Fig 11:**
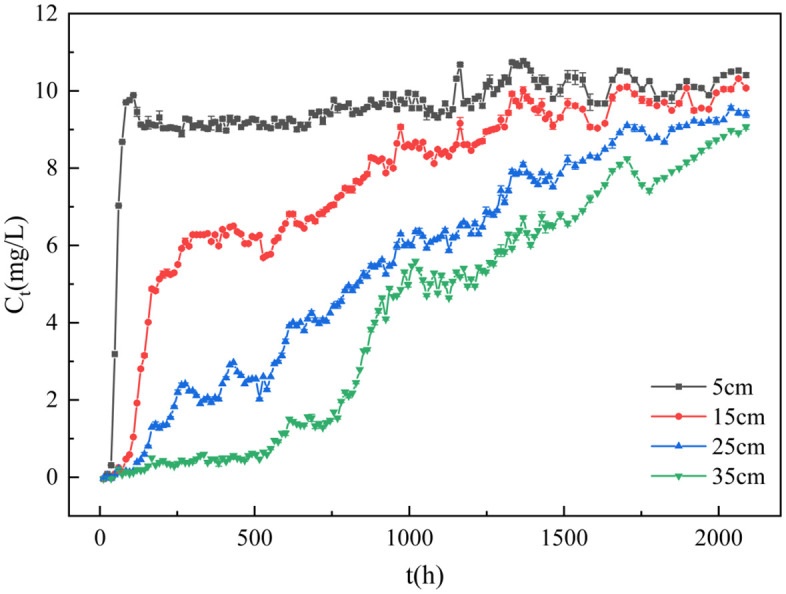
Adsorption breakthrough curve of GT La-WDS.

The sampling ports of the adsorption column were located at elevations of 5 cm, 15 cm, 25 cm, and 35 cm, with corresponding packed particle masses measured as 63.69 g, 179.36 g, 288.20 g, and 390.41 g, respectively. Under room temperature conditions, synthetic phosphorus-containing wastewater was continuously fed through the adsorption column, with breakthrough curves at varying bed heights shown in Fig 11.

As shown in Fig 11, the effluent concentration at the 5 cm bed height rapidly increased during the initial adsorption phase and reached the exhaustion point (C_t_/C_0_ = 0.9). In contrast, the 15 cm bed height exhibited a rapid initial concentration increase followed by a gradual rise, while the 25 cm and 35 cm bed heights demonstrated steady effluent concentration increments throughout the adsorption process. Notably, the exhaustion times (C_t_/C_0_ = 0.9) at 15 cm, 25 cm, and 35 cm bed heights were significantly longer than that at the 5 cm bed height. This phenomenon is attributed to insufficient residence time for phosphate diffusion into the reactive zones of adsorbent particles at lower bed heights, resulting in premature breakthrough (C_t_/C_0_ = 0.1) and exhaustion (C_t_/C_0_ = 0.9) in the 5 cm adsorption column [52]. Furthermore, effluent concentrations at all bed heights exhibited fluctuating upward trends over time. This behavior arises as prolonged contact time enables the phosphate-laden solution to permeate into the interior of upper-layer adsorbent granules. The reactive core regions within these granules become fully activated and participate in phosphate adsorption, generating synergistic effects with lower-layer granules. This multi-scale interaction transiently increases available adsorption sites, thereby enhancing short-term adsorption efficiency. Both breakthrough (C_t_/C_0_ = 0.1) and exhaustion (C_t_/C_0_ = 0.9) points on the breakthrough curves shifted rightward, with breakthrough times increasing from 40 h to 108 h, 162 h, and 588 h, and exhaustion times extending from 84 h to 1272 h, 1704 h, and 2088 h, respectively. This enhancement is attributed to the increased bed height, which expands the accessible reactive adsorption sites within the column, thereby amplifying the phosphorus adsorption capacity of the granules.

Experimental results demonstrate that GT La-WDS achieved a total phosphorus uptake performance of 9.276 mg/g in the 35 cm fixed-bed adsorption system. Effluent phosphate concentrations remained below the China Grade 1B total phosphorus discharge limit (1 mg/L) for 588 h, while sustained phosphorus removal efficiency persisted over 2088 h, validating its long-term operational stability in practical applications. These results demonstrate the high phosphorus removal potential of GT La-WDS as a constructed wetland filler, highlighting its broad applicability in engineered wetland systems.

#### Breakthrough models for dynamic adsorption.

**Fig 12:**
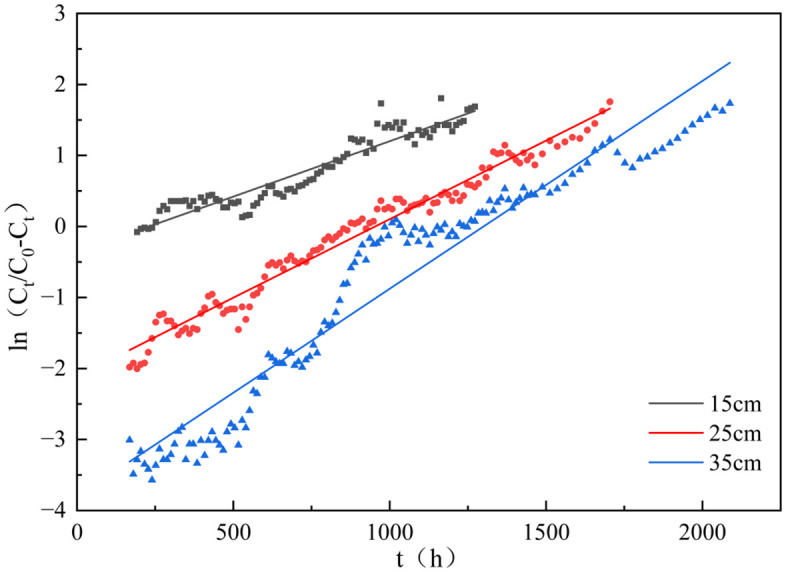
Yoon-Nelson model fits the curve of GT La-WDS.

This study applied the Yoon-Nelson theoretical framework to correlate dynamic adsorption behaviors observed in fixed-bed systems operating at varying column depths (15, 25, 35 cm). The calculation formula of the Yoon-Nelson model is shown in Table S4 in S1 File, while the fitting curves and corresponding parameters are presented in Fig 12 and Table 6, respectively. As shown in Table 6, both the model rate constant K_Y_ and the time τ required for 50% adsorbate adsorption increased with rising bed height. This enhancement can be attributed to the increased density of adsorption binding sites on the granules and extended contact time between the phosphate solution and adsorbent, which synergistically improve phosphorus removal efficiency [53]. Furthermore, the relative error between experimental (τ_exp_) and theoretical (τ_cal_) 50% breakthrough times exceeded 10% for the 15 cm bed height, whereas errors at 25 cm and 35 cm remained below 10%. This discrepancy further corroborates insufficient contact time for phosphate diffusion into the reactive core regions of granules at lower bed heights, resulting in a leftward shift of τ_exp_ (premature breakthrough) in the 15 cm column. However, the Yoon-Nelson model exhibited high correlation coefficients (R_Y_^2^ > 0.9) across all three bed heights, confirming its applicability in describing phosphate adsorption dynamics in these systems [54]. This model enables accurate prediction of time-dependent effluent concentrations, thereby establishing a robust theoretical framework for scaling up GT La-WDS-based adsorption columns in practical engineered wetland applications.

**Table 6:** Yoon-Nelson model parameters.

Sampling hatch	Z(cm)	Penetration point(h)	Exhaustion point(h)	Yoon-Nelson parameters			
				K_Y_ × 10^−3^(1/h)	τ(h)		R_Y_^2^
					Experimental	Calculated	
**1**	15	108	1272	1.6	192	224.3	0.9008
**2**	25	162	1704	2.3	840	930.6	0.9710
**3**	35	588	2088	3.5	1212	1208.4	0.9286

### Conclusions

This study synthesized a granulated lanthanum-modified waterworks-derived sludge composite (GT La-WDS) via calcination-free granulation and systematically investigated its phosphate adsorption performance and mechanisms in aqueous systems. Key conclusions are as follows:

1. Compared to WDS, GT La-WDS demonstrated significant improvements in specific surface area, pore structure, and surface functional groups. Notably, the incorporation of La and Ca further enhanced its phosphate adsorption performance. XRD and FTIR analyses revealed that Al, La, and Ca in GT La-WDS predominantly exist in amorphous forms, thereby effectively preserving the reactive species responsible for phosphate adsorption. Under acidic conditions (pH 4), GT La-WDS demonstrated optimal phosphate adsorption performance, achieving a maximum adsorption capacity of 20.11 mg/g. The adsorption process adhered to the pseudo-second-order kinetic model and Freundlich isotherm, indicating a chemisorption-dominated mechanism occurring on heterogeneous surfaces via multilayer interactions.
2. Phosphate adsorption onto GT La-WDS is primarily mediated by three mechanisms: electrostatic attraction, ligand exchange, and chemical precipitation. Under acidic conditions, phosphate ions formed inner-sphere complexes with Al and La on GT La-WDS surfaces through synergistic electrostatic attraction and ligand-exchange mechanisms. Under alkaline conditions, Ca leaching facilitated hydroxyapatite precipitation via reaction with phosphate, further enhancing phosphorus sequestration efficiency. Phosphorus fractionation analysis demonstrated that the adsorbed phosphorus on GT La-WDS predominantly existed as calcium-linked phosphorus (HCl-P), followed by residual phosphorus (Res-P) and aluminum-complexed phosphorus (NaOH-P), highlighting the critical roles of Ca, La, and Al in phosphorus immobilization mechanisms.
3. Dynamic adsorption column experiments demonstrated that GT La-WDS achieved a total phosphorus uptake performance of 9.276 mg/g in the 35 cm fixed-bed adsorption system. Effluent phosphate concentrations remained below the China Grade 1B total phosphorus discharge limit (1 mg/L) for 588 h, while stable and sustained phosphorus removal efficiency persisted over 2088 h, validating its long-term operational reliability in practical wastewater treatment applications. The breakthrough and exhaustion times were prolonged with increasing column depth of the adsorption column. Furthermore, the Yoon-Nelson model fitting results demonstrated that the dynamic phosphate adsorption process by GT La-WDS was well-described by this model. These findings highlight the high phosphorus removal potential of GT La-WDS as a constructed wetland filler, validating its practical applicability in engineered wastewater treatment systems.

## Supporting information

S1 File Table S1. Determination methods for particle performance indicators. Table S2. The extraction of phosphorus in its various forms. Table S3. The reference method for determining the point of zero charge (pH_pzc_) of GT La-WDS. Table S4. Yoon-Nelson adsorption model formula.
(DOCX)
