# Supplementary material for: Enhanced phosphorus elimination from aquatic systems employing modified granular waterworks derived sludge composite materials: Mechanistic evaluation and process optimization
Source: PLoS One. 2025 Oct 31;20(10):e0334439. doi: 10.1371/journal.pone.0334439 (PMC12578328; doi:10.1371/journal.pone.0334439)
Supplement: S1 File — Table S1. Determination methods for particle performance indicators. Table S2. The extraction of phosphorus in its various forms. Table S3. The reference method for determining the point of zero charge (pHpzc) of GT La-WDS. Table S4. Yoon-Nelson adsorption model formula. (DOCX) [file pone.0334439.s001.docx]

**Supporting information**

**Table S1. Determination Methods for Particle Performance Indicators.**

| **Description** | **Procedure** |
| --- | --- |
| **Breakup rate** | 10 g of ceramsite was dried to constant weight at 105 °C, and 100 mL of water was added. The beaker was placed on a magnetic stirrer and stirred for 1 h. The stirred UCWTR was dried to constant weight and weighed to calculate the mass difference. The percentage of mass loss was determined to characterize the disintegration rate. |
| **Total of breakage and wear rates** | Weigh out 100 g of particles with a size greater than 0.5 mm. Add 6 bearing steel balls (radius 4 mm) to a metal cylinder (radius 25 mm, height 150 mm). Securely close the cylinder lid and agitate it for 15 minutes. After agitation, measure the mass of UCWTR particles smaller than 0.5 mm, and calculate the combined fracture-attrition rate (sum of breakage rate and wear rate). |

**Table S2. The extraction of phosphorus in its various forms.**

| **Step** | **Description** | **Procedure** |
| --- | --- | --- |
| **1** | **NH_4_Cl-P** | The weight of the substrate should be weighed into a centrifuge tube, after which 25 mL of an NH_4_-Cl solution (1 M) should be added. The solution should then be placed in a water-bath oscillator (25 °C and 200 rpm) for a period of 2 h, after which centrifugation should be performed in order to determine the concentration of phosphorus in the supernatant; |
| **2** | **BD-P** | The residue after extraction in step 1 was added to a mixture of 25 mL (0.11 M NaHCO_3_ and 0.11M Na_2_S_2_0_4_), and then put into a water-bath oscillator (25 °C and 200 rpm) to react for 1h, and centrifuged to obtain the supernatant, and then determined the concentration of phosphorus in the supernatant after 24h of resting; |
| **3** | **NaOH-P** | The residue after extraction in step 2 was added with 25 mL of 1 M NaOH solution, and then put into a water-bath oscillator (25 °C and 200 rpm) for 16 h. The concentration of phosphorus in the supernatant was determined by centrifugation; |
| **4** | **HCl-P** | The residue after extraction in step 3 was added with 25 mL of 1 M HCl solution, and then put into a water-bath oscillator (25 °C and 200 rpm) for 16 h. The concentration of phosphorus in the supernatant was determined by centrifugation; |
| **5** | **Res-P** | Following the completion of step 4, 25ml of a 1M NaOH solution should be added to the residue. The centrifuge tube should then be sealed and placed in an oven at 85 °C for one hour. Upon completion of the reaction, the tube should be removed from the oven and subjected to centrifugation. The concentration of phosphorus in the resulting supernatant should then be determined. |

**Table S3. The reference method for determining the point of zero charge (pH_pzc_) of GT La-WDS.**

| **Step** | **Procedure** |
| --- | --- |
| **1** | A 50 mL sodium chloride (NaCl, 0.01 M) solution was placed into 100 mL conical flask with a stopper. 0.01 M HCl and NaOH were used to adjust the successive initial solution pH values from 2 to 12, and 0.25 g of the GT La-WDS sample was added to the conical flask. |
| **2** | The conical flask was filled with N_2_ to eliminate the effect of carbon dioxide (CO_2_) on the pH change, and then the mixture was placed in an oscillator at 303 K for 48 h reaction. |
| **3** | The difference between the final pH and the initial pH, denoted as ΔpH, was plotted against the initial pH. The solution pH at which the curve crossed the line of ΔpH =pH(final)-pH(initial)= 0 was taken as the pH_pzc_ of sample. |

**Table S4. Yoon-Nelson adsorption model formula.**

| **Formula** | **Explanation** |
| --- | --- |
| 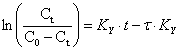 | In the formula, K_Y_ is the model rate constant (h^-1^); τ is the time required to adsorb 50% of the target pollutant (h); C_t_ is the effluent concentration at time t (mg/L); C_0_ is the influent concentration at time t (mg/L). |
